# Tuning the Surface Morphologies and Properties of ZnO Films by the Design of Interfacial Layer

**DOI:** 10.1186/s11671-017-2301-8

**Published:** 2017-09-26

**Authors:** Yaping Li, Hui-Qiong Wang, Hua Zhou, Damin Du, Wei Geng, Dingqu Lin, Xiaohang Chen, Huahan Zhan, Yinghui Zhou, Junyong Kang

**Affiliations:** Fujian Provincial Key Laboratory of Semiconductors and Applications, Collaborative Innovation Center for Optoelectronic Semiconductors and Efficient Devices, Department of Physics, Xiamen University, Xiamen, 361005 People’s Republic of China

**Keywords:** ZnO, Surface morphology, Initial growth, MBE, RHEED, XRD, PL, 81, 81.05.Dz, 81.15.Hi

## Abstract

Wurtzite ZnO films were grown on MgO(111) substrates by plasma-assisted molecular beam epitaxy (MBE). Different initial growth conditions were designed to monitor the film quality. All the grown ZnO films show highly (0001)-oriented textures without in-plane rotation, as illustrated by in situ reflection high-energy electron diffraction (RHEED) and ex situ X-ray diffraction (XRD). As demonstrated by atomic force microscopy (AFM) images, “ridge-like” and “particle-like” surface morphologies are observed for the ZnO films grown in a molecular O_2_ atmosphere with and without an initial deposition of Zn adatoms, respectively, before ZnO growth with oxygen plasma. This artificially designed interfacial layer deeply influences the final surface morphology and optical properties of the ZnO film. From room-temperature photoluminescence (PL) measurements, a strong defect-related green luminescence band appears for the ZnO film with a “particle-like” morphology but was hardly observed in the films with flat “ridge-like” surface morphologies. Our work suggests that the ZnO crystallinity can be improved and defect luminescence can be reduced by designing interfacial layers between substrates and epilayers.

**Electronic supplementary material:**

The online version of this article (10.1186/s11671-017-2301-8) contains supplementary material, which is available to authorized users.

## Background

ZnO is an important semiconductor for optoelectronic applications due to its wide band gap (3.37 eV) and high exciton binding energy (60 meV) [1]. Various ZnO nanostructures, such as 1D nanobelts [2], nanowires [3], nanopoints [4], nanorods [5], nanocables and nanotubes [6], 2D nanowalls [7], and 3D nanotowers [8], have been successfully synthesized. The morphologies and optoelectronic properties of ZnO nanostructures and ZnO films are controlled by adjusting their preparation conditions [8–27]. The crystallinities and morphologies of ZnO-based films have been the focus of many studies [9, 11, 12, 15, 16, 19, 21, 23–25], as these properties play important roles in device applications. Most ZnO films, including those grown by PLD [12], molecular beam epitaxy (MBE) [24], MOCVD [25], and MS [13, 21, 22], have shown “particle-like” surface morphologies. Unconventional surface morphologies, such as networked nanostructures, nanosheets, columnar nanorods [28], and maize corn seed-like morphologies [29], have also been reported. In 2009, Sekine et al. reported that ZnO films with nanoridge surface morphologies showed a high power conversion efficiency improvement of approximately 25% over similar solar cells consisting of planar ZnO nanoparticle films [19]. Banal et al. investigated the formation mechanism of this ridge structure in an AlN/sapphire system and found that the AlN ridge structure formed because of the enhanced migration of Al atoms by an alternating source supply [30]. In addition to the surface structures, the crystallinities and optoelectronic properties of ZnO films have been discussed in several studies [9, 11, 22, 29, 31–33], in which, doping, adding buffer layers, and post-annealing have been reported to be beneficial to improve the properties of ZnO films. In this work, we report the formation of ridge-structured ZnO films by designing an extra interfacial layer and tailoring the initial growth conditions in MBE on MgO(111) substrates. These characteristics have been seldom observed previously for ZnO films grown by MBE. MgO(111) was chosen as a substrate due to its similar hexagonal structure to the ZnO(0001) plane. Additionally, MgO(111) is often used as a buffer layer for high-quality ZnO growth [32, 33].

## Methods

The MgO(111) substrates were cleaned by ultrasonication in acetone and ethanol and subsequently dried with nitrogen before inserting into the MBE growth chamber under ultra-high vacuum. All the substrates were annealed in an oxygen plasma with a partial pressure of 5 × 10^−5^ mbar and a power of 250 W at 490 °C for 60 min. Then, a series of ZnO films were grown with different initial growth conditions, with the detailed growth parameters listed in Table 1 and Additional file 1. Here, step (a) is the thermal treatment of the substrate, and step (b) refers to the growth of the first buffer layer (BLI) without plasma, an important step to change the surface morphology. In previous reports, low-temperature (LT) buffer-layer techniques, which can reduce atom diffusion at the interface and hinder the overflow of Mg atoms from the substrate into the subsequent high-temperature (HT) growth [18, 34], have been employed to improve the crystallinities of ZnO films grown by MBE [15, 33, 35]. Thus, a combination of LT growth in step (c) serves as the second BL (BLII) after the initial growth, and in this work, the thickness of the LT layer is approximately 5 nm. HT growth is utilized for further ZnO film growth, as shown in step (d). According to the different surface morphology features, the specimens are named ZnO particles (ZnO-P) and ZnO ridges (ZnO-R1 and ZnO-R2). The ZnO-P film was grown without BLI, the ZnO-R1 film was grown under the same conditions but with the insertion of BLI in the growth process in the initial stage, and the ZnO-R2 sample was grown using a modified process, also with a BLI, as listed in Table 1. In situ reflection high-energy electron diffraction (RHEED) was used to examine the surface structures of the MgO substrate (before depositing ZnO) and the ZnO films (after the deposition). The surface morphologies and roughnesses were characterized by ex situ AFM and SEM. The growth orientations and crystallinities of the films were further determined by XRD using a Cu anode (*K*_α1_ = 1.54056 Å). In addition, their optoelectronic properties were probed by photoluminescence (PL) measurements.

**Table 1:** Detailed growth conditions for the ZnO films

Samples	Growth processes and detailed parameters
ZnO-P	*a) T* = 490 °C, P(O_2_) = 5 × 10^−5^ Torr, O-plasma = 250 W, *t* = 60 min *b*) ------ *c) T* = 250 °C, P(O_2_) = 1 × 10^−5^ Torr, O-plasma = 180 W, Zn = 330 °C, *t* = 5 min *d) T* = 420 °C, P(O_2_) = 1 × 10^−5^ Torr, O-plasma = 180 W, Zn = 330 °C, *t* = 90 min
ZnO-R1	*a) T* = 490 °C, P(O_2_) = 5 × 10^−5^ Torr, O-plasma = 250 W, t = 60 min *b) T* = 315 °C, P(O_2_) = 5 × 10^−5^ Torr, T(Zn) = 310 °C, *t* = 30 min *c) T* = 250 °C, P(O_2_) = 1 × 10^−5^ Torr, O-plasma = 180 W, Zn = 330 °C, *t* = 5 min *d) T* = 420 °C, P(O_2_) = 1 × 10^−5^ Torr, O-plasma = 180 W, Zn = 330 °C, *t* = 90 min
ZnO-R2	*a) T* = 490 °C, P(O_2_) = 5 × 10^−5^ Torr, O-plasma = 250 W, *t* = 60 min *b) T* = 370 °C, P(O_2_) = 5 × 10^−5^ Torr, T(Zn) = 340 °C, *t* = 30 min *c) T* = 250 °C, P(O_2_) = 5 × 10^−5^ Torr, O-plasma = 200 W, Zn = 340 °C, *t* = 5 min *d) T* = 490 °C, P(O_2_) = 5 × 10^−5^ Torr, O-plasma = 200 W, Zn = 340 °C, *t* = 60 min

*T* temperature (°C), *P* oxygen partial pressure (mbar), *plasma* oxygen plasma power (W), *t* time (min)

## Discussion

**Fig. 1:**
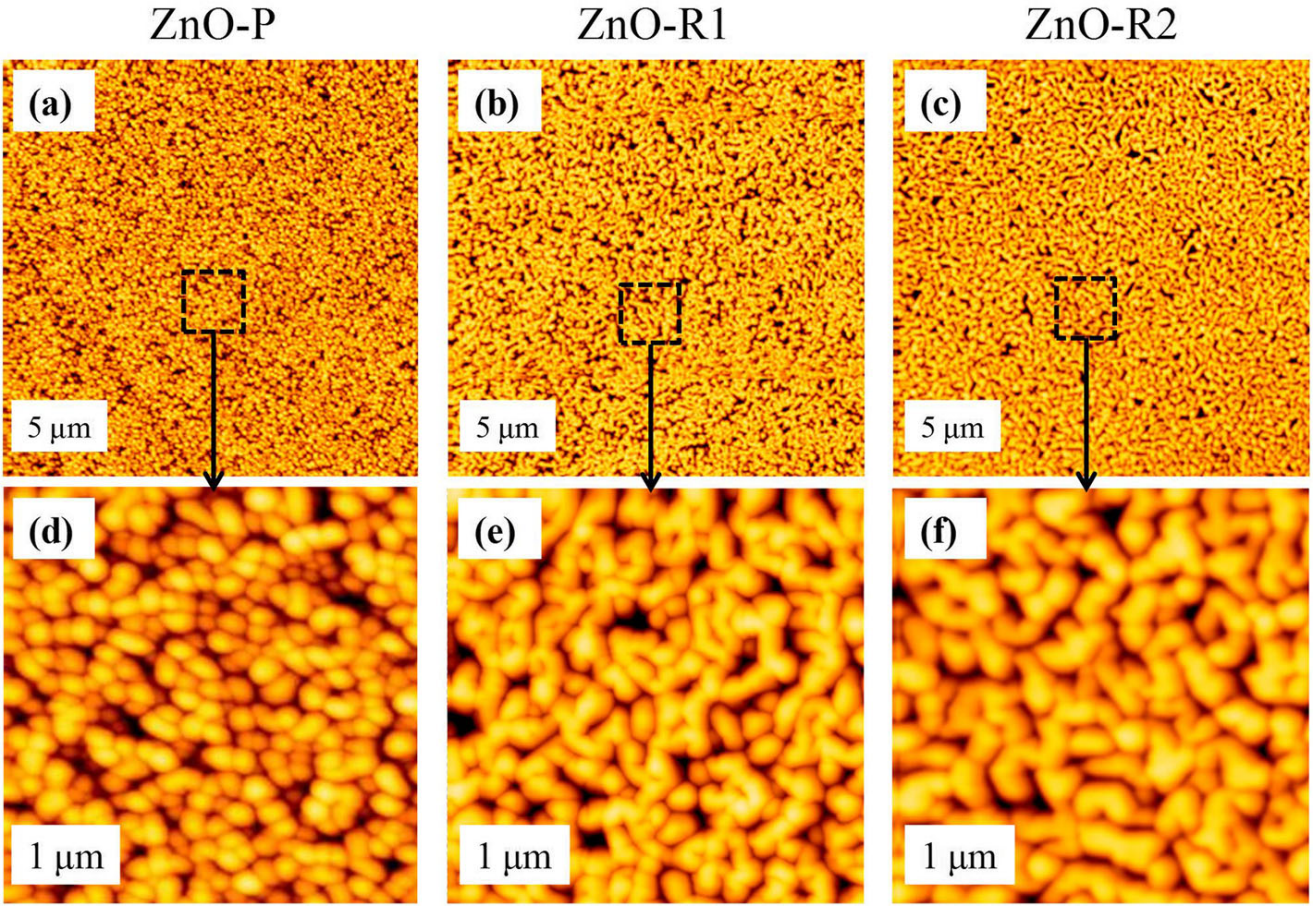
AFM results. **a**–**c** AFM images of the ZnO film surface morphologies (5 μm). **d**–**f** Magnified images of the square areas (marked by dashed black lines) in **a**–**c**

The surface morphologies of the ZnO films with different growth conditions were studied by AFM. The inserted interfacial layer had an important influence on the surface morphologies of the thin films. In Fig. 1a, the AFM image of the ZnO-P film shows a distribution of nanoparticles. On the other hand, the AFM images of both the ZnO-R1 and ZnO-R2 films show more ridge-like features, as shown in Fig. 1b, c. Figure 1d–f shows the magnified images of the square area (marked by dashed black lines) in Fig. 1a–c. The mean particle diameter of ZnO-P in Fig. 1d is approximately 70 nm, and the mean ridge width of ZnO-R1 in Fig. 1e is approximately 70 nm, with the existence of many apertures among the ridges. For the modified ZnO-R2 sample, the ridges are more compact and wider than those in ZnO-R1, displaying a mean width of 90 nm and less holes among the ridges. The surface roughnesses are further confirmed by the root-mean-square (RMS) values of 4.15, 7.51, and 3.10 nm for the ZnO-P, ZnO-R1, and ZnO-R2 films, respectively. In our specimens, BLI plays an important role in the morphology. A series of samples with different substrate temperatures with BLI were prepared, which all display ridge-like surface morphologies, but some samples possess surface defects, as shown in Additional file 1. Based on the comparison of the films with and without BLI, the initial nucleation of ZnO was found to determine the ultimate specific morphology. Additionally, the oxygen pressure also played a very important role in the nucleation process, which showed high sensitivity, as Zn atoms could easily desorb without surrounding oxygen due to their low adhesion energy [36, 37]. This special ridge morphology is somewhat similar to that of a previous report [38], in which a particle-like morphology composed of 3D columnar grains was transformed to a nanoridge morphology after a 30-min HT post-annealing, which drove the lateral coalescence of the grains. However, in this work, lateral coalescence occurs during the growth stages. Similar to the initial nucleation of AlN [30], the Zn atoms prefer to migrate to special step edges of the substrate, followed by combination with O_2_ to form ZnO at the edges, even though O_2_ is not activated by plasma, thus forming the ridge-like morphology. Surface migration of adatoms during the initial growth stage (an extremely flat surface) would result in high-quality ZnO crystals. On the other hand, without BLI, the ZnO film is deposited directly onto the substrate surface with O activated by plasma, resulting in a typical nanoparticle surface morphology. Therefore, the interfacial layer, which is primarily determined by the initial growth process, is the main factor leading to the final ZnO morphology. Our results are similar to those of previous studies reporting that the inserted BL incites grain coalescence in the films [11, 31]. In addition, the HT process could facilitate ZnMgO formation at the interface of ZnO and MgO via the diffusion of Zn and Mg atoms into the MgO substrate and ZnO film [37, 39] and further evaporation [38]. SEM was also performed to characterize the surface morphologies of the ZnO thin films, as shown in Additional file 1: Figure S2. The two SEM images of the ZnO films with typical particle and ridge-like surface morphologies display similar results to those from AFM.

**Fig. 2:**
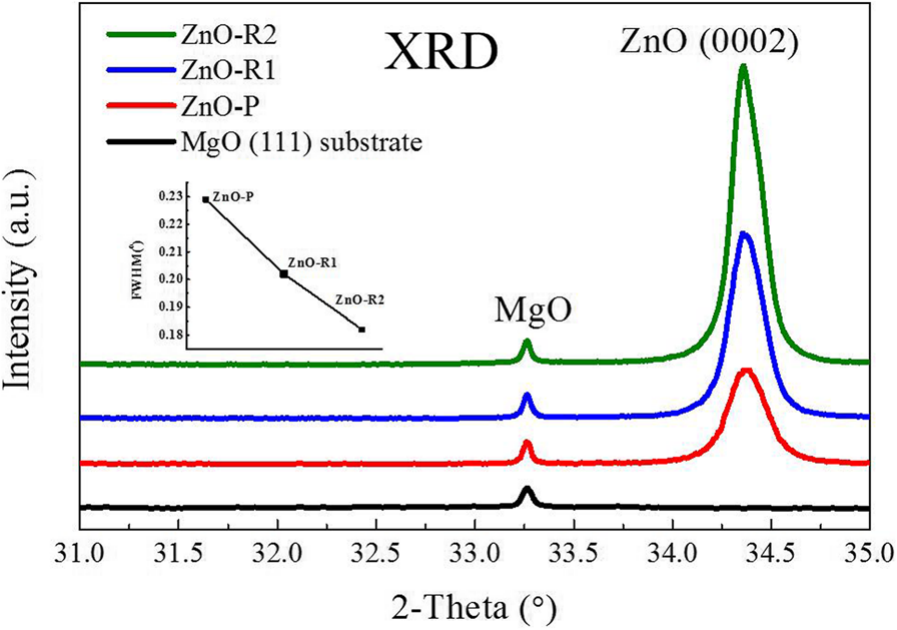
XRD results. XRD patterns of the MgO(111) substrate and films with particle- or ridge-like morphologies. The inset shows the FWHMs of the ZnO(0002) peak for these three specimens

Figure 2 shows the XRD results of the ZnO films grown with and without BLI. Only one ZnO peak was observed for all three specimens, indicating highly (0001)-oriented textual structures. The positions of the ZnO(0002) peaks ranged from 34.36° to 34.38°, displaying smaller shifts compared to that of bulk ZnO (34.4°). In this work, the peak positions were located at 34.38°, 34.37°, and 34.36° for ZnO-P, ZnO-R1, and ZnO-R2, respectively. According to the Scherrer equation, 2*d*sin*θ* = *jλ*, the lattice constants along the *c* axis were calculated to be larger than that of bulk ZnO, indicating that these films exhibit tensile stress along the *c* axis. Two possible factors affecting the lattice strain are illustrated by the variation of the (0002) diffraction peak position: (1) the lattice mismatch between the ZnO film and the MgO(111) substrate and (2) the existence of point defects (vacancies and interstitial atoms) caused by the growth conditions, such as Zn-rich or oxygen-rich conditions [40]. The intensities of (0002) peaks for ZnO films were normalized using the MgO substrate peak at 33.26°. The ZnO(0002) peak intensity of ZnO-P is obviously weaker than those of ZnO-R1 and ZnO-R2. In addition, the FWHM values for ZnO-P, ZnO-R1, and ZnO-R2 are 0.229, 0.202, and 0.182, respectively, as shown in the upper-left inset of Fig. 2. The FWHM value is associated with the dislocation density [11, 41], with a larger value indicating the possibility of more dislocations in the films. Therefore, the ridge-like ZnO films show better crystallization than the particle-like films, indicating that the lateral coalescence of small grains greatly improve the crystallinity of ZnO films, which is consistent with previous results [11, 14, 15, 31]. As temperature is one of the most important growth parameters, the BLI growth temperature was tuned from 250 to 450 °C, and the optimal temperature was found to be 315 °C. Similar to the AFM results, unsuitable temperature leads to poor crystallinity and optical properties (discussed below). The ZnO(0002) peak intensity decreases when the temperature is too low (such as 250 °C) or too high (such as 450 °C), as shown in Additional file 1.

**Fig. 3:**
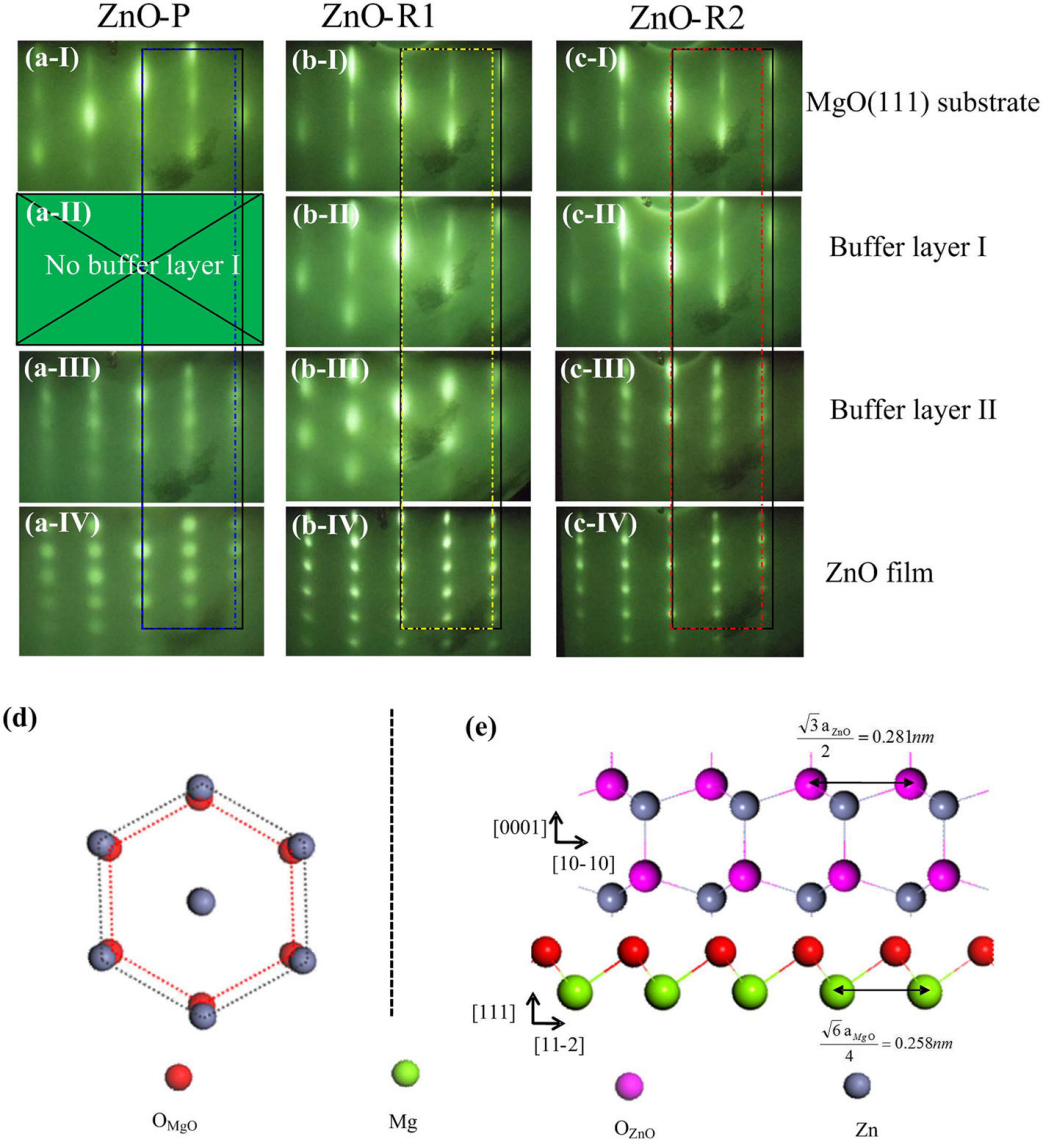
RHEED results and structure models. **a**–**c** RHEED patterns of the surface structures for the substrate and the epilayers recorded at different stages (I, II, III, IV). **d**, **e** Schematic models of the epitaxial relationship between the MgO(111) substrate and ZnO(0001) epilayers

The evolution of the sample surface structure during the growth process was monitored using in situ RHEED. The RHEED patterns of these three grown ZnO films exhibit spotty features for either a particle-like or ridge-like surface morphology, as shown in Fig. 3. The pattern of the substrate after thermal treatment shows streaky features (Fig. 3a-I, b-I, c-I), indicating the presence of a flat surface, and the distance between stripes corresponds to an in-plane lattice constant of 0.298 nm for the MgO(111) plane. Without BLI in ZnO-P, the mixing of spots and stripes indicates that ZnO grains nucleate on the substrate surface after the LT growth of BLII. In addition, these patterns can be used to deduce the lattice spacing, assuming that the MgO(111) in-plane lattice constant equals the bulk value of 2.98 Å. Thus, the distance between stripes becomes narrower as the in-plane lattice constant transitions from MgO to ZnO, as shown in Fig. 3a-I, a-IV. However, as the blue short dash-dot line indicates, after 90 min of growth, the in-plane lattice constant in the ZnO-P film remains similar to that after the LT growth of BLII, i.e., larger than that in bulk ZnO. Thus, in-plane strain may exist in the film. This situation almost vanishes for the other two films with BLI. Even with dotted patterns, the in-plane lattice constants for these two ZnO films are very close to that in the bulk sample. From the RHEED patterns after 30 min of BLI growth, as shown in Fig. 3b-II, c-II, the patterns remain streaky, indicating relatively flat surfaces. Furthermore, the distance between these stripes are slightly smaller than that in the substrate but obviously larger than that of ZnO, which could be the result of ZnMgO interfacial layers due to the diffusion of Zn atoms into the MgO(111) substrate [37, 42]. Upon completing in 5 min the LT growth of BLII, the streaky pattern completely disappears and becomes spotty, as shown in Fig. 3b-III, c-III, indicating a 3D island growth model of the ZnO film at the initial stage. This observation agrees with a previous report that found the aggregation of adatoms results in the formation of 3D islands [43]. In addition, the in-plane lattice constants are larger than those in Fig. 3b-II, c-II but still smaller than those of the thick ZnO films shown in Fig. 3b-IV, c-IV. These results show that, upon deposition of BLII, the ZnO films are deposited but residual stress still exists. This stress is completely relaxed after the subsequent HT growth. The patterns of the ridge-like ZnO films after HT growth demonstrate better crystallinity compared to that of the particle-like ZnO films. A model of the epitaxial relationship between the MgO(111) substrate and ZnO film is illustrated in Fig. 3d, e: ZnO [1–210]//MgO [1–10] and ZnO [1–100]//MgO [11–2]. The lattice mismatch value was calculated to be (3.25 − 2.98)/2.98 = 9%, which agrees well with our RHEED results.

As previously reported, the ZnO growth orientation can be altered by different growth conditions or substrates [15, 27, 39]. In this work, the use of hexangular MgO(111) substrates leads to a single growth orientation, which is consistent with the previous expression of the rotational symmetries of the substrate and epilayer, as determined by the formula [44]: $N=\frac{\mathit{\operatorname{lcm}}\left(n,m\right)}{C_m}$, where *N* denotes the number of rotation domains in the epilayer; *n* and *m* denote the rotational symmetries of the substrate (MgO(111) plane) and epilayer (ZnO(0001) plane), respectively; and *lcm*(*n*,*m*) denotes the least common multiple of *n* and *m*. Both the MgO(111) substrate and wurtzite ZnO film possess sixfold symmetry; thus, only one ZnO domain exists on the substrate. This result coincides with the results of the RHEED patterns and XRD spectra in this work.

**Fig. 4:**
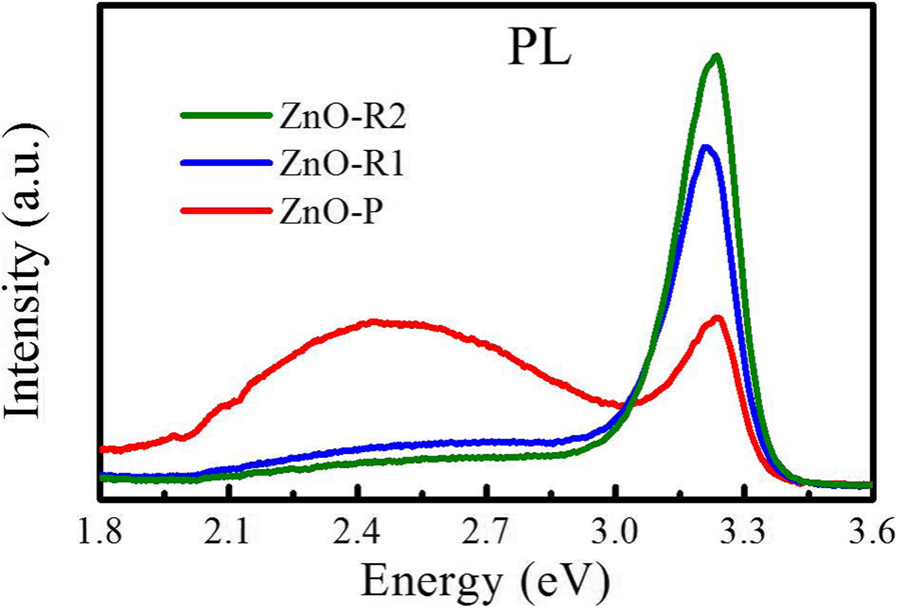
PL results. Room-temperature PL spectra of the ZnO films with particle- or ridge-like morphologies

The optoelectronic properties of the ZnO epilayers were investigated by room-temperature PL measurements, as shown in Fig. 4. The PL spectra of all the ZnO films contain a strong band-edge transition emission at approximately 3.23 eV, which is redshifted from that in the bulk ZnO, and this shift is related to the changed bandgap of the ZnO films. Previous reports have indicated that lattice mismatch between ZnO and sapphire could persist even in a film as thick as 1 μm, leading to a redshift of 50 meV for the band-edge emission peak [45, 46]. In addition, variations of the surface morphology and oxygen vacancy population are also the factors that cause this change [47]. The PL emissions of the two ridge-like films exhibit much stronger intensities with FWHMs of 123 and 133 meV for ZnO-R1 and ZnO-R2, respectively, which are smaller than that of the particle-like sample and smaller than that of a ZnO film grown on the (111) plane of cubic YSZ [48]. Particularly, a green emission band appears at approximately 2.5 eV in ZnO-P, which is similar to that of a ZnO film deposited on MgO(100) [49]. In general, oxygen vacancies [50], surface morphology [47, 51], and oxygen clusters formed on the surface [52] are major origins for the green emission band. ZnO films with densely vertically aligned ZnO nanorods have been reported to possess stronger green emission bands relative to films with small particle and nanosheet morphologies [47]. Additionally, the stronger visible emission band likely originates from the abundant surface defects and surface states of the thin films with larger specific surface area. Zhan et al. [50] proposed the presence of two sub-bands centered at 2.14 and 2.37 eV, which correspond to unoccupied oxygen vacancies and singly occupied oxygen vacancies [53, 54], respectively. Babu et al. [34] proposed that oxygen vacancy (V_O_) and zinc interstitial (Zn_i_) created by the diffusion of Mg atoms at the interface of ZnO and MgO enhance the green emission, which is in good agreement with theoretical predictions [55]. This green emission band is much weaker in ZnO-R1 and ZnO-R2, which could be due to the interfacial BLI that makes Zn-rich conditions. The Zn adatoms can consume the oxygen atoms absorbed on the substrate to form ZnO. However, the ZnO-P sample is fabricated without BLI, leaving the oxygen clusters on the substrate surface and thus generating a strong green emission band. Therefore, both the oxygen vacancies and surface state may be responsible for the green emission band, and as an artificially inserted interfacial layer, BLI helps prevent the diffusion of Mg atoms from the substrate into the subsequently deposited ZnO epilayers, hence further reducing the green emission band.

## Conclusion

In this paper, ZnO films with ridge-like surface morphologies, which were found to be sensitive to the initial oxygen pressure, were prepared on MgO(111) substrates and were compared to a traditional particle-like ZnO film. A series of experiments were performed to investigate the factors influencing the morphology. In situ RHEED measurements confirmed that all the grown ZnO thin films showed a wurtzite phase. In addition, interfacial layers were proposed to form between the substrates and epilayers. ZnO films with neat ridge-like surface features exhibited favorable crystallinities and optoelectronic properties compared to those of the ZnO film with a particle-like surface structure. Our work suggests that the surface morphology, film crystallinity, and emission properties could be highly improved by inserting an artificial interfacial layer. ZnO films with ridge-like structures could promote the application of ZnO in lasers, vacuum fluorescent or field emission displays, high-power and high-frequency devices, light-emitting diodes, etc.

## Additional file

Additional file 1: Supplementary experimental data for ZnO films grown on MgO (111). **Table S1.** Detailed growth conditions for ZnO film samples. **Figure S1.** AFM results. (a)-(e) AFM images of the ZnO film surface morphologies in 5μm; (f)-(j) magnified images of the square areas (marked by dashed black lines) in (a)-(e). **Figure S2.** SEM results. SEM images for ZnO films with typical particle and ridge surface morphologies. **Figure S3.** XRD results. XRD plots for MgO (111) substrate and films. **Figure S4.** PL results. Room temperature PL spectra of ZnO films. (PDF 88 kb)

## Abbreviations

AFM: Atomic force microscopy
BL: Buffer layer
FWHM: Full width at half maximum
HT: High temperature
LT: Low temperature
MBE: Molecular beam epitaxy
MOCVD: Metal organic chemical vapor deposition
MS: Magnetron sputtering
PL: Photoluminescence
PLD: Pulsed laser deposition
RHEED: Reflection high-energy electron diffraction
SEM: Scanning electron microscopy
XRD: X-ray diffraction
