# Supplementary material for: Tuning the Surface Morphologies and Properties of ZnO Films by the Design of Interfacial Layer
Source: Nanoscale Res Lett. 2017 Sep 26;12:551. doi: 10.1186/s11671-017-2301-8 (PMC5615082; doi:10.1186/s11671-017-2301-8)
Supplement: Additional file 1: — Supplementary experimental data for ZnO films grown on MgO (111). Table S1. Detailed growth conditions for ZnO film samples. Figure S1. AFM results. (a)-(e) AFM images of the ZnO film surface morphologies in 5μm; (f)-(j) magnified images of the square areas (marked by dashed black lines) in (a)-(e). Figure S2. SEM results. SEM images for ZnO films with typical particle and ridge surface morphologies. Figure S3. XRD results. XRD plots for MgO (111) substrate and films. Figure S4. PL results. Room temperature PL spectra of ZnO films. (PDF 88 kb) [file 11671_2017_2301_MOESM1_ESM.pdf]

ADDITIONAL FILE

Tuning the surface morphologies and properties of ZnO films by the design of interfacial layer

Yaping Li, Hui-Qiong Wang*, Hua Zhou, Damin Du, Wei Geng, Dingqu Lin, Xiaohang Chen, Huahan Zhan, Yinghui Zhou, Junyong Kang

Fujian Provincial Key Laboratory of Semiconductors and Applications, Collaborative Innovation Center for Optoelectronic Semiconductors and Efficient Devices, Department of Physics, Xiamen University, Xiamen 361005, P.R. China

*Corresponding author. Email: hqwang@xmu.edu.cn

A series of ZnO films with different BL1 deposition temperatures were prepared to study the relationship between the final ridge-like morphology and the BL1 growth temperature. ZnO film grown without oxygen in the BLI growth step was also prepared to investigate the role of oxygen for preparation of the special ridge-like morphology. Detailed preparation conditions for the ZnO films with different temperatures and oxygen atmospheres in the BLI growth step are shown in Table S1. In this table, samples of ZnO without BLI (ZnO-P in main text) and ZnO-315°C (ZnO-R in main text) are represented for comparison.

Table S1 Detailed growth conditions for the ZnO films

Samples	Growth processes and detailed parameters
ZnO-without BLI (ZnO-P)	a) T=490°C, $P(O_2)=5\times10^{-5}$ mbar, O-plasma=250 W, t=60 min;<br>b) ------<br>c) T=250°C, $P(O_2)=1\times10^{-5}$ mbar, O-plasma=180 W, Zn=330°C, t=5 min;<br>d) T=420°C, $P(O_2)=1\times10^{-5}$ mbar, O-plasma=180 W, Zn=330°C, t=90 min.
ZnO-No O_2 in BLI	a) T=490°C, $P(O_2)=5\times10^{-5}$ mbar, O-plasma=250 W, t=60 min;<br>b) T=315°C, $P=2.7\times10^{-7}$mbar, T(Zn)=310°C, t=30 min;<br>c) T=250°C, $P(O_2)=1\times10^{-5}$ mbar, O-plasma=180 W, Zn=330°C, t=5 min;<br>d) T=420°C, $P(O_2)=1\times10^{-5}$ mbar, O-plasma=180 W, Zn=330°C, t=90 min.
ZnO-250°C	a) T=490°C, $P(O_2)=5\times10^{-5}$ mbar, O-plasma=250 W, t=60 min;<br>b) T=250°C, $P(O_2)=5\times10^{-5}$ mbar, T(Zn)=310°C, t=30 min;<br>c) T=250°C, $P(O_2)=1\times10^{-5}$ mbar, O-plasma=180 W, Zn=330°C, t=5 min;<br>d) T=420°C, $P(O_2)=1\times10^{-5}$ mbar, O-plasma=180 W, Zn=330°C, t=90 min.
ZnO-315°C (ZnO-R1)	a) T=490°C, $P(O_2)=5\times10^{-5}$ mbar, O-plasma=250 W, t=60 min;<br>b) T=315°C, $P(O_2)=5\times10^{-5}$ mbar, T(Zn)=310°C, t=30 min;<br>c) T=250°C, $P(O_2)=1\times10^{-5}$ mbar, O-plasma=180 W, Zn=330°C, t=5 min;<br>d) T=420°C, $P(O_2)=1\times10^{-5}$ mbar, O-plasma=180 W, Zn=330°C, t=90 min.

ZnO-450°C	a) T=490°C, $P(O_2)=5\times10^{-5}$ mbar, O-plasma=250 W, t=60 min;<br>b) T=450°C, $P(O_2)=5\times10^{-5}$ mbar, T(Zn)=310°C, t=30 min;<br>c) T=250°C, $P(O_2)=1\times10^{-5}$ mbar, O-plasma=180 W, Zn=330°C, t=5 min;<br>d) T=420°C, $P(O_2)=1\times10^{-5}$ mbar, O-plasma=180 W, Zn=330°C, t=90 min.

T= temperature (ºC), $P(O_2)$= oxygen partial pressure (mbar), plasma= oxygen plasma power (W), t=time (min)

Figure S1shows the AFM images with different BL1 growth conditions. The AFM images of the film without BLI (ZnO-P in main text) and the film grown with BLI at 315°C (ZnO-R1in main text) are shown in Figure S1. The film without oxygen in the BLI growth shows a particle-like morphology, which indicates that both oxygen and zinc atoms are essential for the special ridge-like morphology. In addition, all the films show ridge-like morphologies with different BL1 growth temperature, but some surface defects appear with unsuitable temperatures (e.g. 250°C and 450°C). Based on this comparison of samples, we concluded that the interfacial layer between the MgO substrate and ZnO film ultimately determines the specific morphology formed.

Figure S2 shows the SEM images of two typical samples with particle- and ridge-like surface morphologies, respectively. The ZnO film in S2(a) is composed of lots of small particles, while the ZnO film in S2(b) consists of dense and packed ridges, showing two typical surface morphologies of particles and ridges, respectively. These results are similar to those obtained from AFM.

The XRD patterns of the ZnO films with different BLI deposition conditions are shown in Figure S3. The film without BLI (ZnO-P in main text) and the film grown with BLI at 315°C replotted for comparison. All the films grew along the c axis, and the crystallinity was roughly deduced from the width of the peak, from which the film grown with BLI at 315°C (ZnO-R1 in main text) shows the best crystallinity.

The PL spectra of the ZnO films with different BLI deposition conditions are shown in Figure S4. The film without BLI (ZnO-P in main text) and the film grown with BLI at 315°C (ZnO-R1) are replotted for comparison. According to the PL spectra, a band-edge transition emission is shown at approximately 3.23eV in all the films, and an additional green emission band can also be observed for all the films, except the film growth at 315°C with BLI. As discussed in the main text, this band is primarily related to the defects in the films. Similar to the results from XRD, 315°C is an optimal temperature for BL1 growth.

Figure Captions

Figure S1 AFM results. (a)-(e) AFM images of the ZnO film surface morphologies (5μm). (f)-(j) Magnified images of the square areas (marked by dashed black lines) in (a)-(e).

Figure S2 SEM results. SEM images of the ZnO films with typical particle- (a) and ridge- (b) like surface morphologies.

Figure S3 XRD results. XRD patterns of the MgO(111) substrate and films.

FigureS4 PL results. Room-temperature PL spectra of the ZnO films.
